# Early versus Late Admission to Labor Affects Labor Progression and Risk of Cesarean Section in Nulliparous Women

**DOI:** 10.3389/fmed.2016.00026

**Published:** 2016-06-27

**Authors:** Rafael T. Mikolajczyk, Jun Zhang, Jagteshwar Grewal, Linda C. Chan, Antje Petersen, Mechthild M. Gross

**Affiliations:** ^1^Department for Epidemiology of Infectious Diseases, Hannover Medical School, Hannover, Germany; ^2^Department of Epidemiology, Helmholtz-Centre for Infection Research, Braunschweig, Germany; ^3^Shanghai Key Laboratory of Children’s Environmental Health, Xinhua Hospital, Shanghai Jiaotong University School of Medicine, Shanghai, China; ^4^Epidemiology Branch, Eunice Kennedy Shriver National Institute of Child Health and Human Development, National Institutes of Health, Bethesda, MD, USA; ^5^Maternal-Fetal Medicine, Department of Obstetrics and Gynecology, Naval Hospital Camp Lejeune, Camp Lejeune, NC, USA; ^6^Midwifery Research and Education Unit, Department of Gynecology and Obstetrics, Hannover Medical School, Hannover, Germany

**Keywords:** cesarean section, Kaplan-Meier life table analysis, oxytocin, dilation, early admission, timing of interventions

## Abstract

**Background:**

Rates of cesarean section increase worldwide, and the components of this increase are partially unknown. A strong role is prescribed to dystocia, and at the same time, the diagnosis of dystocia is highly subjective. Previous studies indicated that risk of cesarean is higher when women are admitted to the hospital early in the labor.

**Methods:**

We examined data on 1,202 nulliparous women with singleton, vertex pregnancies and spontaneous labor onset. We selected three groups based on cervical dilatation at admission: early (0.5–1.5 cm, *N* = 178), intermediate (2.5–3.5 cm, *N* = 320), and late (4.5–5.5 cm, *N* = 175). The Kaplan–Meier estimator was used to analyze the risk of delivery by cesarean section at a given dilatation, and thin-plate spline regression with a binary outcome (R library *gam*) to assess the form of the associations between the cesarean section in either the first or second stage versus vaginal delivery and dilatation at admission.

**Results:**

Women who were admitted to labor early had a higher risk of delivery by cesarean section (18 versus 4% in the late admission group), while the risk of instrumental delivery did not differ (24 versus 24%). Before 4 cm dilatation, the earlier a woman was admitted to labor, the higher was her risk of delivery by cesarean section. After 4 cm dilatation, however, the relationship disappeared. These patterns were true for both first and second stage cesarean deliveries. Oxytocin use was associated with a higher risk of cesarean section only in the middle group (2.5–3.5 cm dilatation at admission).

**Conclusion:**

Early admission to labor was associated with a significantly higher risk of delivery by cesarean section during the first and second stages. Differential effects of oxytocin augmentation depending on dilation at admission may suggest that admission at the early stage of labor is an indicator rather than a risk factor itself, but admission at the intermediate stage (2.5–3.5 cm) becomes a risk factor itself. Further research is needed to study this hypothesis.

## Introduction

The high and steadily increasing rates of cesarean section both in the US (1–4) and worldwide (5) are a cause for concern and provide motivation to study the underlying causes of these patterns. One potential explanation is the higher prevalence of medical interventions during labor, including a more liberal diagnosis of dystocia (6–10). Despite considerable efforts to define and standardize dystocia, there still remains substantial variety regarding the diagnosis of onset of labor, especially if it is taken into account that women usually diagnose labor onset by themselves (11, 12).

Meanwhile, several studies have determined that cesarean rates are higher among women admitted during the early stages of labor (i.e., with early cervical dilatation) than among those who were admitted at a later stage (i.e., with advanced cervical dilatation) (13–28). The reasons for this association are not clear. For example, the higher cesarean rates among women admitted during the early stages of labor could result from false labor and the subsequent pressure to expedite delivery (29, 30). In fact, early oxytocin augmentation is more likely among those admitted early (27, 31), and while it can hasten labor and prevent labor arrest in some cases (32), in others, it can lead to exhaustion and subsequent labor arrest (33, 34). Similarly, the early initiation of analgesia could make a longer labor more tolerable, but it can also be an indicator of underlying problems and might therefore be associated with increased probability of a cesarean section (35, 36). On the other side, early admission and the accumulation and cascade of following interventions itself can be an indicator of higher risk for abnormal labor (37–39).

In addition to observational studies, evidence is available from evaluations of triage programs that have been introduced to limit admission during the early stages of labor or reduce its effects (13, 17, 27, 40). The studies generally demonstrated only minor, respective non-significant effects of interventions, but could not explain this observation.

So far, the mechanism by which early admission increases the risk of cesarean section remains unclear, providing necessity for further studies. While there is consensus that risk of cesarean is elevated in women with early admission, the previous studies did not assess this association in a longitudinal fashion during the labor progression and in detail allowing addressing potential non-linearity in the expected effects. For example, whether the higher risk is only related to first stage cesarean section or whether it is accumulated in early stages of labor, indicating overall poor progress of labor. Therefore, we aimed at assessing the association between cervical dilatation at admission and labor progression as well as the risk of cesarean section during the first and second stages of labor and its interplay with oxytocin augmentation.

## Materials and Methods

### Sample

The analysis employs data collected for a previous study assessing the impact of an increase in the rates of epidural analgesia and the risk of cesarean section, the details of which are published elsewhere (41). In brief, two samples of 500–700 women each were selected from two time periods – one prior to an introduction of the policy advocating the use of epidural analgesia for all women during labor in a military hospital in Hawaii (1992/1993) and the other after the introduction (1995/1996). The study was limited to nulliparous women between 18 and 34 years of age at admission, with singleton, vertex pregnancies where the gestational age, based on the last menstrual period or ultrasound, was estimated to be between 37 0/7 and 41 6/7 weeks at delivery. These women had experienced a spontaneous onset of labor and were able to give birth to infants that weighed between 2,500 and 4,000 g. A woman was ineligible for the study if either her dilatation upon admission was >7 cm or labor duration from admission until birth was <3 h, as there would potentially be insufficient time for the administration of an epidural analgesia. In total, 1,329 women were recruited for the original study; however, for the purposes of our analysis, we used data from 1,202 women for whom records of dilatation at admission were available.

Trained midwives reviewed the medical records of the study participants to obtain information on demographic characteristics, assessment of cervical dilatation at admission, labor progression, and administration of any analgesia, birth, and postpartum circumstances.

This study was approved by the Institutional Review Boards of the National Institute of Child Health and Human Development, National Institutes of Health, and the Tripler Army Medical Center. As the analysis was based on medical records only, no informed consent was required.

### Statistical Analysis

First, we tabulated the maternal characteristics and variables related to labor for the whole sample and for three groups based on the extent of dilatation at admission: (1) 0.5–1.5 cm, (2) 2.5–3.5 cm, and (3) 4.5–5.5 cm. These three non-adjacent ranges were selected, so as to avoid classification problems at cut-off values. The first two groups fall within the latent phase of labor, whereas the third is classified as the active phase, based on the criterion of more than 4 cm for the active phase which was valid at the time when the research was performed (42). Differences across groups were tested by Wilcoxon and chi-square test. Second, we assessed the relationship between the extent of dilatation at admission and the risk of cesarean section during the first and second stages employing thin-plate spline regression (R library *gam*). This permitted the inspection of the form of the association (43). In this analysis, the total sample was included. Subsequently, we used the Kaplan–Meier life table analysis to study the patterns of oxytocin augmentation and cesarean section over the course of labor in the three subgroups defined by dilatation at admission. Vaginal birth was considered as the end of the studied period, and labor progress was expressed in terms of dilation. In the analysis of the oxytocin augmentation, first stage cesarean was considered as a censoring event. Differences between groups were tested by log-rank test. Finally, we studied the probability cesarean section in the subgroups of dilatation at admission comparing women who received oxytocin and those who did not.

## Results

**Figure 1:**
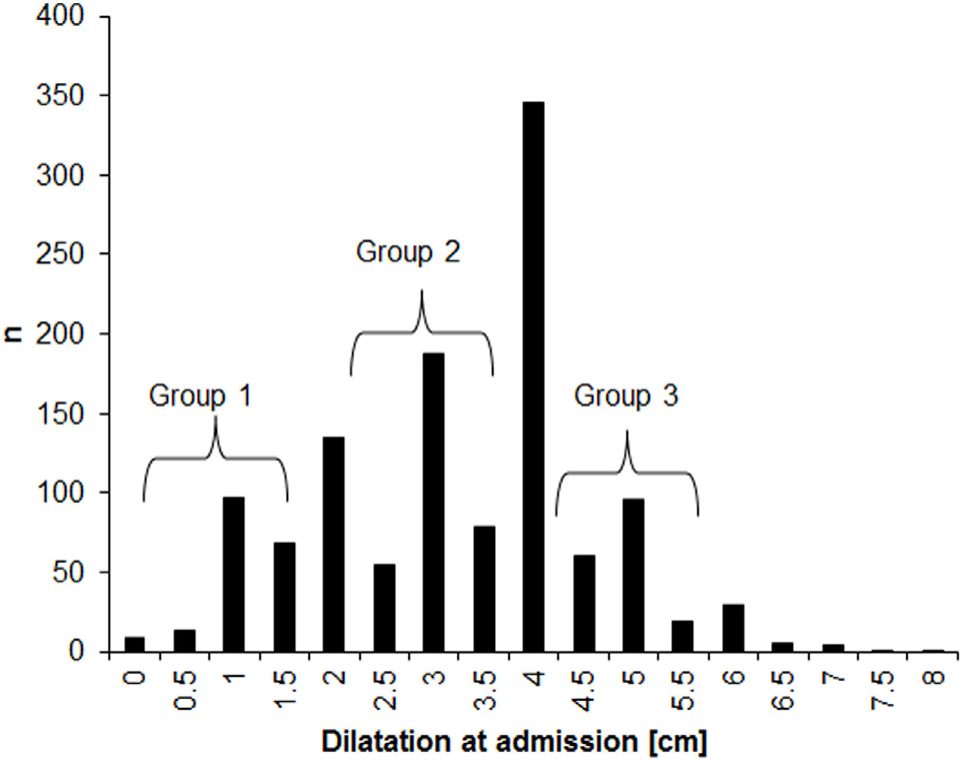
**Distribution of dilatation at admission in the sample (*n* = 1,202)**.

The distribution of dilatations at admission for the 1,202 participants who were included in the analysis is presented in Figure 1. About half (53%) of the women were admitted with dilatations below 4 cm. There was clear rounding tendency, with dilatation much more often ending in full centimeter than in 0.5 cm. This rounding tendency supports our decision to analyze subgroups based on non-adjacent dilatation ranges in order to minimize the misclassification error. No differences in maternal age, BMI, and birth weight were observed across the three subgroups of dilatation at admission (Table 1). The oxytocin augmentation and cesarean sections were significantly more frequent among women with <3.5 cm dilatation at admission, as compared with among those with dilatations between 4.5 and 5.5 cm. The risk of instrumental vaginal delivery did not vary across the three groups.

**Table 1:** **Characteristics of the study subjects by dilatation at admission**.

	Total sample	Groups defined on the basis of cervical dilatation at admission			
	*N* = 1202	0.5–1.5 cm *N* = 178	2.5–3.5 cm *N* = 320	4.5–5.5 cm *N* = 175	*p*-Value^a^
**General characteristics**					
Maternal age (years) (mean, SD)	23.6 (3.8)	24.3 (4.0)	23.4 (3.7)	23.5 (3.7)	0.029
Body mass index (kg/m^2^) (mean, SD)	22.5 (3.7)	22.1 (3.7)	22.7 (3.9)	22.6 (3.9)	0.21
Birth weight (g) (mean, SD)	3385 (336)	3328 (339)	3415 (322)	3385 (323)	0.015
**Labor and delivery**					
Oxytocin augmentation	53%	77%	56%	30%	<0.0001
Epidural analgesia^b^	53%	49%	50%	50%	0.95
**Delivery mode**					
Cesarean section	13%	18%	15%	4%	0.0002^c^
Instrumental vaginal birth	22%	24%	23%	23%	0.98^c^
Spontaneous vaginal birth	65%	58%	62%	73%	0.015^c^

*^a^Chi-squared or Wilcoxon tests comparing cervical dilatation at admission groups*.

*^b^Epidural analgesia was strongly influenced by the change in the hospital policy and does not reflect individual preferences or risks*.

*^c^Each outcome tested versus the complementary category: cesarean section versus all other, spontaneous vaginal birth versus cesarean section and instrumental vaginal birth*.

**Figure 2:**
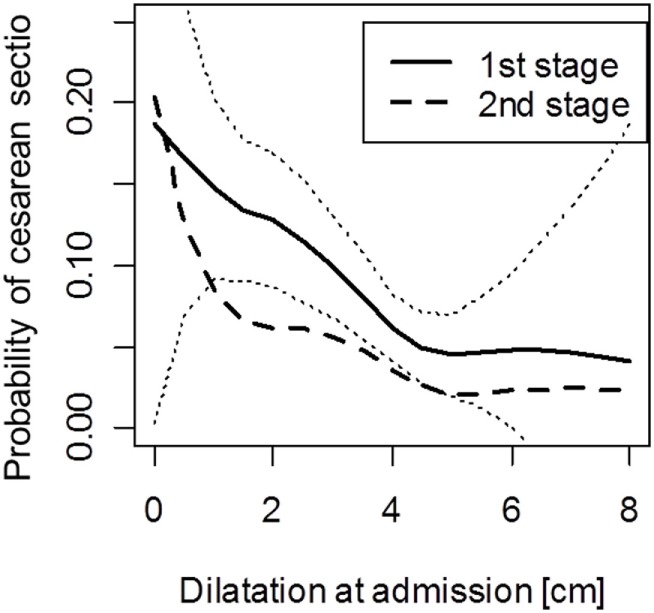
**Probability of cesarean section during the first and second stages of labor for women with a different dilatation at admission**. Note: analysis for second stage excludes women who had cesarean in the first stage. Dotted lines indicate pointwise 95% confidence intervals for the first stage cesarean delivery.

The risk of first and second stages cesarean section decreased approximately linearly with the dilatation at admission for dilatations below 5 cm and was constant for dilatations above 5 cm (Figure 2). The data for women with >5 cm dilatation, however, were sparse, and the results may therefore be unreliable, as indicated by the wide confidence bands (Figure 2).

**Figure 3:**
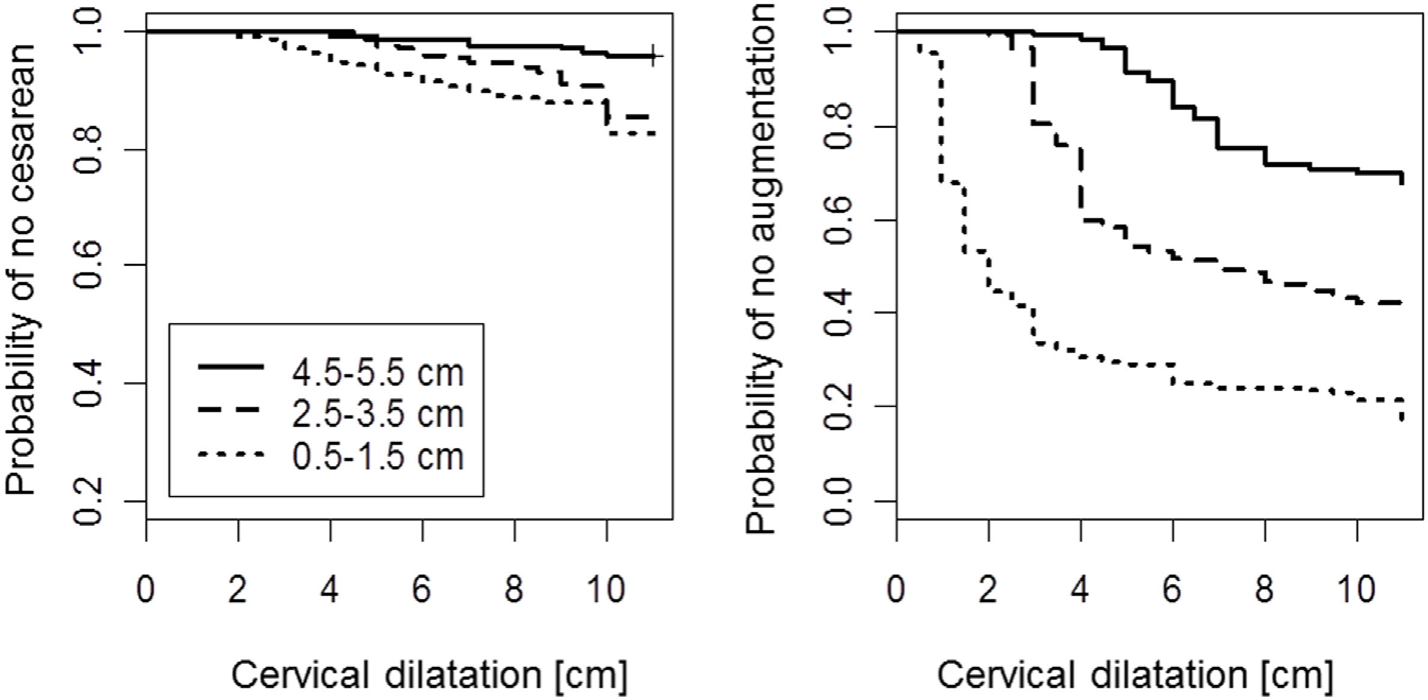
**Kaplan–Meier survival curves for cesarean delivery (left) and initiation of oxytocin augmentation (right) by cervical dilatation**. Note: model for augmentation considers women who were delivered by cesarean before augmentation as censored. Second stage is displayed as dilatation of 11 cm.

In all three dilatation subgroups, the risk of cesarean section was constant during labor (as measured by dilation), with higher rates in the early admission groups (Figure 3). As a consequence, among women who were admitted early (i.e., with dilatations of <4 cm) and where a cesarean section before reaching 5 cm dilatation was not conducted, the risk of cesarean at more advanced dilatation was still slightly elevated, as compared with those who were admitted with a dilatation of more than 4 cm (*p* = 0.013 in log-rank test).

By contrast, labor augmentation with oxytocin followed a different pattern. Among most women admitted with <4 cm dilatation, the use of oxytocin was initiated shortly (measured by dilation) after admission (Figure 3, right panel). However, if a woman passed the 5-cm dilation mark without receiving oxytocin, the initiation of oxytocin use did not vary significantly across the three dilatation subgroups (*p* = 0.09 in log-rank test).

In the total sample, oxytocin use was associated with a higher risk of cesarean section (Table 2). In the subgroups defined on the basis of cervical dilatation at admission, there was no association in the groups admitted with 0.5–1.5 cm dilatation and with 4.5–5.5 cm dilatation (in the latter group possibly due to a too small sample). In contrast, there was a strong effect in the middle group (2.5–3.5 cm dilatation at admission).

**Table 2:** **Mode of birth depending on the administration of oxytocin among women admitted with different cervical dilatations**.

	Total sample		Groups defined on the basis of cervical dilatation at admission					
			0.5–1.5 cm		2.5–3.5 cm		4.5–5.5 cm	
	Oxy+ (%)	Oxy− (%)	Oxy+ (%)	Oxy− (%)	Oxy+ (%)	Oxy− (%)	Oxy+ (%)	Oxy− (%)
Vaginal birth	80.9	95.0	83.1	80.5	78.9	93.7	92.5	97.5
Cesarean first stage	12.5	3.4	11.8	12.2	12.6	4.9	7.6	1.6
Cesarean second stage	6.6	1.6	5.2	7.3	8.6	1.4	0	0.8
*p*-Value*	<0.0001		0.86		0.0007		0.11	

*Oxy+/− specifies whether oxytocin was administered during the course of labor*.

*Chi-squared test comparing oxy+ and oxy− groups.

## Discussion

Our results show that the probability of cesarean section was much higher for women admitted early as compared with those admitted late, which is consistent with several previous studies (13–26). We could show that the elevated risk persisted during both the active phase and the second stage of labor and that it was not accumulated during a specific time of labor. Oxytocin augmentation was more frequent among women admitted early, and in most cases, the oxytocin augmentation was initiated shortly after admission (in terms of dilation). For the observed higher risk of cesarean section among women admitted to labor early, there are two potential explanations: first, the risk is pre-existent and early admission is an indicator of problems during the course of labor and, second, the risk increase occurs during the course of labor, with early admission facilitating a cascade of interventions finally resulting in cesarean section (37, 44). For example, some women admitted early may receive interventions like oxytocin augmentation because of the expectation that they should progress in the labor. They may not respond to the labor stimulation. Since these women may not be in true labor, early admission could be resulting in the diagnosis of dystocia and performance of a cesarean section (9, 17). The third possibility is that mixture of both mechanisms become active in the same woman and also that in some women the one mechanism is responsible, while in the others the other mechanism.

The ascertained results allow rejection of some hypotheses regarding the course of labor in women after early admission (for example, we showed that in case of early admission, the risk of cesarean section is not concentrated in the early labor, and that the risk of cesarean is elevated through the first and second stages). A possible mechanism for such concentration of risk in the early labor could be the failure to account for the non-linearity in the dilation process. The risk of cesarean section may be higher when predictions regarding dilation in the early stages of labor are based on what is expected for dilation at a later stage of labor given the different transition times and based on the action lines when a partogram is used (42, 45–47). This would suggest problems in management of labor. Yet, our findings of constant cesarean probabilities across all stages of dilation do not support this hypothesis.

Our finding of frequent oxytocin administration shortly after admission in the early admission group could suggest that labor progress in these women is hampered from the beginning and continue to be such during the complete course of labor (37, 38). However, the early use of oxytocin can also contribute to the observed elevated risk for cesarean section: women who receive oxytocin early in labor may be exhausted by the time they reach the second stage, resulting in the diagnosis of dystocia at this late stage (33). Interesting observation is that oxytocin administration was associated with no change in risk of cesarean among women in earliest and latest studied admission groups, while it was in the intermediate admission group. This suggests a non-linear effect of oxytocin on the mode of birth in the process of labor. Non-linear effects of interventions have been observed regarding labor duration and mode of birth only recently (48). Assuming that oxytocin augmentation is one of the potential elements of the cascade of interventions during labor, the interpretation could be that women in the earliest admission group are those bringing their elevated risk into labor, whereas in the intermediate admission group, there is a contribution of the management of labor to the increased risk. The mechanism in this intermediate admission group might be explained by uterine hyperstimulation, resulting in fetal acidosis as an indication for cesarean section (34, 49). A review analyzing partogram action lines found the lowest incidence of oxytocin augmentation and cesarean sections with a 4-h action line and excluding the latent phase (47). If the women are admitted at a later stage of labor, the oxytocin effects are less pronounced (or at least matter less, because cesarean section is generally less common). Unfortunately, our data did not contain information on the indication for oxytocin augmentation. Further studies are necessary to assess the potentially differential effects of oxytocin administration depending on the dilatation at admission and its association with labor progress. Early administration of oxytocin alone or in combination with amniotomy was shown to shorten labor duration (34). At the same time, reviews found inconsistent results regarding the prevention of cesarean section (32, 34).

Fortunately, taking advantage of data collected during the change in the policy of conducting epidural analgesia, we were able to indirectly control the effects of differential pain perception. For example, a woman may perceive the early stages of labor as painful, and the resulting anxiety could lead to an early admission in the hospital which nowadays is in some places provided with special offers for early labor care (15, 50). A previous study found higher rates and an earlier initiation of epidural analgesia when women perceived their personal onset of labor earlier than their midwife diagnosed the onset of labor (51). Therefore, a low individual pain threshold and a high anxiety level, together with a difficult labor, may result in higher rates of cesarean section (44). In the analyzed data, epidural analgesia that typically can be applied to relieve pain was determined mainly by policy and not by individual preferences/needs of the women.

### Strengths and Limitations

The strength of our study is the homogenous database of nulliparous women and the in-depth analysis of the risk of cesarean during the progress of the labor. Furthermore, as stated above, taking advantage of data collected during a “natural experiment” of epidural analgesia, we were able to leave this variable out from the consideration. The main limitation is the observational nature of this study; since direct causal inference is not possible, we were only able to generate hypotheses for further research, as in relation to oxytocin. Another limitation is the measurement error and subjectivity inherent in measurement of cervical dilation.

## Conclusion

Early admission to labor was associated with a significantly higher risk of cesarean section, which depended on dilatation at admission. This finding is consistent with previous literature and supplements the previous knowledge by demonstrating a constant risk during the labor progress and a risk elevation for first and second stages of labor. The fact that oxytocin administration was associated with the risk of cesarean section depending on dilatation at admission may suggest that admission at the early stage of labor (0.5–1.5 cm) is an indicator rather than a risk factor itself, but it becomes a risk factor when admissions occur in the intermediate stage (2.5–3.5 cm). Future research should address this specific hypothesis in more detailed analyses.

## Author Contributions

RM performed the analysis and drafted the manuscript; JZ conceptualized the research question, performed the original study, and provided comments on the manuscript; JG, LC, AP, and MG contributed to interpretation of the data and the writing of the manuscript. All authors approved the version being submitted.

## Disclaimer

The views expressed in this article are those of the authors and do not necessarily reflect the official policy or position of the Department of the Navy, Department of Defense, nor the United States Government.

## Conflict of Interest Statement

The authors declare that the research was conducted in the absence of any commercial or financial relationships that could be construed as a potential conflict of interest.
